# Development and Validation of an RP-HPLC Method for CB13 Evaluation in Several PLGA Nanoparticle Systems

**DOI:** 10.1100/2012/737526

**Published:** 2012-06-18

**Authors:** J. Álvarez-Fuentes, L. Martín-Banderas, I. Muñoz-Rubio, M. A. Holgado, M. Fernández-Arévalo

**Affiliations:** Department of Pharmacy and Pharmaceutical Technology, Faculty of Pharmacy, University of Seville, C/Profesor García González, 41012 Seville, Spain

## Abstract

A simple, fast, and reversed-phase high-performance liquid chromatographic (RP-HPLC) method has been developed and validated for determining of a cannabinoid derivate, which displays potent antihyperalgesic activity, 1-naphthalenyl[4-(pentyloxy)-1-naphthalenyl]methanone (CB13) into PLGA nanoparticles. Separation was achieved in a C18 column using a mobile phase consisting of two solvents: solvent A, consisting of acetonitrile : water : acetic acid (75 : 23.7 : 1.3 v/v), and solvent B, consisting of acetonitrile. An isocratic method (70 : 30 v/v), with a flow rate of 1.000 mL/min, and a diode array detector were used. The developed method was precise, accurate, and linear over the concentration range of analysis with a limit of detection and a limit of quantification of 0.5 and 1.25 **μ**g/mL, respectively. The developed method was applied to the analysis of CB13 in nanoparticles samples obtained by three different procedures (SEV, FF, and NPP) in terms of encapsulation efficiency and drug release. Nanoparticles size and size distribution were also evaluated founding that NPP method presented the most lowest particle sizes with narrow-size distribution (**≈**320 nm) and slightly negative zeta potential (**≈**−25 mV) which presumes a suitable procedure for the synthesis of PLGA-CB13 nanoparticles for oral administration.

## 1. Introduction

Nowadays, there is a special interest in the development of new delivery systems able to allow an exhaustive control over their physicochemical profiles. Nanotechnology is providing a very useful technological tool in everything related to the development of nanoparticle systems for actives. These nanosystems have become of great interest because of their capability to provide a wide range of products for several administration routes able to exert a temporal and/or spatial control in their release profiles [1–3]. According to the objectives pursued with the use of the pretended nanoparticle systems, there are several key elements that must be considered because of their great influence over the products: (i) the type of technology/method used in the production of the nanoparticles, (ii) the physicochemical properties of the molecules carried in the particles, and (iii) the specifically properties of the polymers employed. 

Related to the active molecules to be carried by these nanosystems, one of the most important researches in medicine is the treatment of pain. The pain reduces the quality of life for millions of patients around the world and drug treatments currently available, normally opioids and anti-inflammatory drugs, are not effective in many clinical situations. Cannabinoids have antinociceptive mechanisms different from those used by the drugs currently employed, providing a new line for the treatment of pain that is unresponsive to drug treatments presently available [4]. Oral administration is one of the routes most commonly used for drug administration. However, it is not feasible when the actives present unfavourable conditions: not adequate physicochemical properties for intestinal absorption, stability or solubility problems, and clear decrease in bioavailability by first-pass hepatic effects, as the cannabinoids [4, 5].

**Figure 1:**
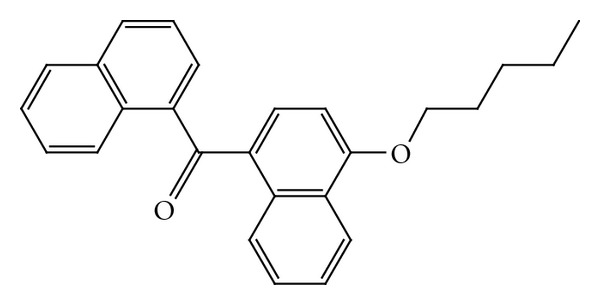
CB13 structural formula (C_26_H_24_O_2_).

A cannabinoid derivate was used in our study (Figure 1): 1-naphthalenyl[4-(pentyloxy)-1-naphthalenyl]methanone (CB13). 

It is a CB1/CB2 dual agonist (selective activation of peripheral cannabinoid CB1 receptors has the potential to become a valuable therapy for chronic pain) which displays potent antihyperalgesic activity in animal models and limited brain penetration [6]. In the other hand, CB13 is a cannabinoid derivate highly lipophilic which belongs to class II compounds, and as a consequence of its poor solubility and dissolution in the gastrointestinal fluids, is incompletely absorbed [7].

During the last three decades, there has been continuous interest in the use of biodegradable polymers for the development of nano- and micropolymeric delivery systems able to improve drugs oral bioavailability, to control their therapeutic effect and to prolong it. Their encapsulation within drug delivery systems allows to have a better pharmacokinetics pathway and to reduce drastically the frequency of injection. In order to successfully develop these formulations, poly(lactic-*co*-glycolic) acid (PLGA), one of the few polymers approved by the Food and Drug Administration for human clinical use, was chosen as biodegradable polymer [8–10]. PLGA polymers have shown to be biocompatible and they degrade to toxicologically acceptable lactic and glycolic acids [11].

In present work the production of different method for CB13 encapsulation into PLGA nanoparticles was carried out: (i) emulsion solvent evaporation method assisted by ultrasounds (SEV-US) [12]; (ii) emulsion solvent evaporation method assisted by Flow Focusing (SEV-FF) [13] (iii) nanoprecipitation (NPP) [14]. Parameters such as particle size, particles size distribution, surface charge, particle morphology, drug loading, and *in vitro* release profile were evaluated.

The main goal of this study was the development and validation of a simple and rapid HPLC method for quantification of this molecule from possible pharmaceutical dosage forms derived from nanoparticle systems. Several other HPLC methods were also developed for the determination of cannabinoids [15–18]. All of these methods, however, are not employed for the determination of CB13 in a possible pharmaceutical dosage form and usually employed to quantify illicit substances in biological fluids. Fischedick et al. [18] developed a HPLC method for cannabinoids quantification extracted from plant material. Mercolini et al. [16] and Abbara et al. [17] developed HPLC methods for the analysis of cannabinoids in urine and plasma after solid-phase extraction.

In order to achieve this purpose, the analytical method proposed has been used for investigating drug loading and *in vitro* release profiles CB13 nanosystems produced by these three different nanoencapsulation techniques. 

## 2. Materials and Methods

### 2.1. Materials

1-Naphthalenyl[4-(pentyloxy)-1-naphtha-lenyl] methanone (CB 13) was obtained from Tocris (UK); HPLC-grade acetonitrile, acetic acid, and ethyl acetate were purchased from Panreac (Spain). Poly(lactic-*co*-glycolic) acid block copolymer (PLGA 50 : 50) Resomer RG 502 (Mw: 12000; inherent viscosity: 0.24 dL/g) was obtained from Boehringer Ingelheim (Germany). Surfactants employed, Span 60, Mowiol 3-96 (PVA), and Pluronic F-68, were obtained from Sigma-Aldrich.

Deionised and purified water using a Milli-Q system (Millipore) was used for the standard solutions preparation. All other reagents were of analytical grade.

### 2.2. RP-HPLC Method: Development and Validation

#### 2.2.1. Equipment and Chromatographic Conditions

The chromatographic apparatus consisted of a Hitachi system manager D-7000, equipped with a quaternary pump L-7100, a diode array detector L-7455, an automatic injector L-7200, and interface D-7000. For data collection and calculation, HSM System Manager Software was used.

The chromatographic conditions [19–21] were a column C18 (Waters Spherisorb 5 *μ*m ODS2; 4.6 × 250 mm Analytical Column, Ireland) and a mobile phase consisting of two solvents: a mixture of acetonitrile : water : acetic acid (A) (75 : 23.7 : 1.3 v/v) and acetonitrile (B). Eluent (70 : 30% A : B) was pumped at 1.000 mL/min. The detection wavelength UV was 230 nm and the injection volume was 10 *μ*L. The operating temperatures were maintained at room temperature but the oven was heated at 40°C to favour the mobile phase flow through the column.

#### 2.2.2. Preparation of Standard and Sample Solutions

Standard stock solution of CB13 at a concentration of 500 *μ*g/mL was prepared by weighting accurately 500 *μ*g of CB13 and dissolving it in  1 mL of acetonitrile. it was shaken vigorously in a vortex until complete solubilization. Then, it was filtered and injected into HPLC. 

To carry out the sample solution, it was accurately weighted around 5 mg of nanoparticles and added 1 mL of acetonitrile. The samples were shaken vigorously in a vortex (5 minutes) to promote the solubilization of cannabinoid from the nanoparticles. BLAG from nanoparticles does not interfere with analyte at the wavelength that CB13 is quantified (see the complete validation study process). The samples were filtered with 0.22 *μ*m nylon-membrane filter (Millipore, Barcelona) and injected directly into HPLC [22].

#### 2.2.3. Validation of the Method

The method was validated in agreement with International Conference on Harmonization (ICH) [23], using the following analytical parameters: linearity, precision, accuracy, specificity, detection and quantification limits, and robustness.

- *Selectivity/specificity*: was determined by comparing nanoparticle carrier samples with and without CB13 (placebo).

In this work, the selectivity of the method was evaluated in three different samples (solution of placebo, solvent, and standard solution of CB13 (500 *μ*g/mL)), which were injected to check their specificity. 

Moreover, degradation studies, where the standard solutions of the drug were subjected to different degrees of stress (ICH), were carried out: temperature, light, and pH. 

CB13 is not soluble in acidic or basic aqueous solutions, so that, first, it was solubilized in its normal solvent (acetonitrile) and then, it was acidified or alkalinized with a little amount of acid or base and it was incubated for 5 hours. Later, the solutions were neutralized with basic or acid solutions and completed to a final volume with acetonitrile; for heat-forced degradation, a standard solution of CB13 was incubated 24 h in 60°C (oven); for sun light forced degradation, a standard solution of CB13 was exposed 24 h to sun light.

After the stress assay, the samples were analyzed by HPLC as shown in the chromatographic conditions.

(ii) *Precision*: was assessed by testing the repeatability of three different standard solutions 10 times in the same day (intra-day) and by intermediate precision analyzing the same three standard solutions on different days (*n* = 10) (inter-day).
(iii) *Accuracy*: was tested by mean percentage recoveries of three samples of CB13 at five different concentrations precisely prepared and by determination of the relative standard deviation (RSD). Specificity was determined by comparing nanoparticulate carrier samples with and without CB13 (placebo).

It was studied the concentration levels of 50%, 75%, 100%, 125%, and 150%, where a known amount of the active was added to a determined amount of placebo solution to obtain drug concentrations of 250, 375, 500, 625, and 750 *μ*g/mL, respectively. The amount of CB13 recovered in relation to the added amount (recovery percent), was calculated [23].

(iv) *Linearity*: a linear relationship should be evaluated across the range of the analytical procedure. It was demonstrated directly on the drug substance (by dilution of a standard stock solution).

Linearity should be evaluated by visual inspection of a plot of signals as a function of analyte concentration or content. If there is a linear relationship, test results should be evaluated by appropriate statistical methods. For the establishment of linearity, a minimum of 5 concentrations is recommended [24].

This study was performed by evaluating the system and method linearity. For the system linearity, standard solutions of CB13 at five concentrations levels, from 50% at 150% of the target analyte concentration, were calculated. Each level of concentration was prepared in triplicate. The experimental results were graphically plotted, obtaining a calibration curve and carrying out the corresponding statistical study.

For the method linearity, the procedure was the same as that of system linearity, but the sample was a solution containing the PLGA nanoparticles (placebo) and adding an increased amount of CB13, dissolved in the medium. The results were treated the same way for the system linearity [20].

#### 2.2.4. Limit of Detection (LOD) and Quantification (LOQ)

LOD and LOQ tests for the procedure were performed on samples containing progressively more dilute concentrations of analyte. Afterwards, the concentrations *versus* the RSD obtained for area from each of the concentrations were plotted in order to determine LOD and LOQ [25].

#### 2.2.5. Robustness

The robustness/ruggedness of an analytical procedure is a measure of its capacity to remain unaffected by small but deliberate variations in method parameters and provides an indication of its reliability during normal usage [23] according to the application.

In the case of liquid chromatography, examples of these variations are changes in pH of the mobile phase (±0.2 units); variation in mobile phase composition (±8% of each solvent); oven temperature (±2°C) and flow rate (±0.1 mL/min) [26].

The pH adjustment procedure was carried out in agreement with International Conference on Harmonization (ICH) [24], using solutions of hydrochloric acid and sodium hydroxide.

### 2.3. Preparation of CB13-Loaded Nanoparticles

In present work three different methods for CB13 nanoparticles synthesis were assayed. CB13 is a cannabinoid derivate highly lipophilic which belongs to class II compounds (low solubility and a high permeability) of the Biopharmaceutics Classification System (BCS), showing low water solubility (~0.001–0.002 mg/mL). As a consequence of its poor solubility and dissolution in the gastrointestinal fluids, this compound is incompletely absorbed [7]. Although there are diverse strategies (use of cosolvents, salt formation, complexes with cyclodextrins, etc.) to solve this problem, various nanotechnology-based drug delivery systems have emerged to increase the bioavailability of numerous drugs that are poorly soluble in water [27, 28]. So, to improve CB13 oral bioavailability, it was incorporated into PLGA nanoparticles by three methods.

***Emulsion-Solvent Evaporation Method Assisted by Ultrasounds *(SEV-US) [29]**

An o/w emulsion was prepared to obtain solid PLGA nanoparticles. As oil phase a cosolution of cannabinoid (0.1 mL, 0.5% w/v) and PLGA (1 mL, 10% w/v) in ethyl acetate (EA) was prepared. This solution was added dropwise to a 0.3% (w/v) PVA solution under sonication. The recently prepared emulsion was diluted by adding 20 mL of a 2% (w/v) PVA solution, stirred at r.t. for 4 h. After this, particles were collected by centrifugation (10000 rpm, 4°C, and 20 min) and washed three times with distilled water. Finally, particles were freezing dried (Cryodos, Telstar) and stored at 4°C.

***Emulsion-Solvent Evaporation Method Assisted by Flow-Focusing* (SEV-FF) [30]**

In this case, to prepare the o/w emulsion, a simple Flow Focusing nozzle (Ingeniatrics Tecnologías, Spain) was used. As oil phase (focused fluid), a cosolution of cannabinoid (0.1 mL, 0.5% w/v) and PLGA (1 mL, 10% w/v) in ethyl acetate (EA) was injected at 0.2 mL/h. As aqueous phase (focusing fluid), a distilled water was injected at 2 mL/min. The o/w emulsion is collected inside a PVA (0.5% w/v) bath under magnetic agitation at r.t. for 4 h. After this, particles were manipulated as previously described [31].

***Nanoprecipitation* (NPP) [32]**

Briefly, a co-solution of PLGA (1.5% w/v), CB13 (0.25% w/v), and Span 60 (0.5% w/v) in acetone was dropped onto a Pluronic F68 (0.5% w/v) solution at 5 mL/min flow rate under magnetic stirring. After acetone evaporation, NPs suspension was filtered by 1 *μ*m pore size filter (Millipore). After this, particles were manipulated as previously described.

### 2.4. Characterization Methods

The mean diameter and size distribution of CB13 loaded-PLGA nanoparticles were measured at 25.0 ± 0.5°C by a laser scattering technique based on Mie theory (Partica LA-950V2, Horiba, Japan).

Nanoparticles surface charge was determined by zeta potential (ZP) measurements. The ZP of the particles was determined by laser Doppler (Zetamaster 300, Malvern Instruments Ltd, Malvern, UK). ZP measurements were carried out in triplicate after washing the nanoparticles with distilled water at r.t.

The shape and morphology characteristics of the nanoparticles were determined by scanning electron microscopy (SEM) (Philips XL-30, USA) after coating lyophilised samples with a gold thin film.

### 2.5. Evaluation of Drug Content from PLGA Nanoparticles

CB13 content of nanoparticles was assessed directly by HPLC from the extraction of the drug of nanoparticles. The drug content was expressed as encapsulation efficiency percentage (EE%) and drug loading (%) following (1):

(1) $$\begin{array}{cc} & \text{EE\%}\\ & \;=\left(\frac{\text{actual}\;\text{amount}\;\text{of}\;\text{CB}13\;\;\text{loaded}\;\text{in}\;\text{NP}}{\text{theory}\;\text{amount}\;\text{of}\;\text{CB}13\;\;\text{in}\;\text{NP}}\right)\times 100,\\ & \text{Drug}\;\text{loading}\;\left(\text{\%}\;\frac{\text{w}}{\text{w}}\right)\\ & \;=\left(\frac{\text{mass}\;\text{of}\;\text{CB}13\;\text{in}\;\text{NPs}}{\text{mass}\;\text{of}\;\text{NP}\;\text{recovered}}\right)\times 100. \end{array}$$

### 2.6. **In Vitro** Release Profile

To establish the CB13 release profile from nanoparticles at simulated gastric and intestinal pH, nanoparticles were suspended in USP XXVI HCl buffer pH 1.2 or USP XXVI HCl buffer pH 6.8 at 37°C and stirred mechanically (100 rpm) during the release experiments (Unitronic OR, Selecta, Spain). Aliquots (500 *μ*L) were withdrawn at fixed time intervals and filtered upon centrifugation at 8000 rpm. The filtered sample (Millex GV) (10 *μ*L) was injected into the HPLC apparatus for the evaluation of CB13.

## 3. Results

### 3.1. HPLC Method Development

The chromatographic conditions were optimized for the determination of CB13 within a suitable analysis time (20 min) and peak (isolated, symmetric, etc.). 

With regard to the mobile phase, an HPLC method for cannabinoids was described [19], in which methanol, water and acetic acid (75 : 23.7 : 1.3, v/v/v) were used as the mobile phase. However, the time retention was too longer and a major proportion of methanol could decrease the CB13 retention time. So, the following mobile phase was a mix 70 : 30 (v/v) of two solutions: the solution given above and a methanol solution. Later, methanol was changed to acetonitrile because the first produced gas in the HPLC system and the peak moved to different retention times. 

### 3.2. Validation Study

#### 3.2.1. Selectivity/Specificity

**Figure 2:**
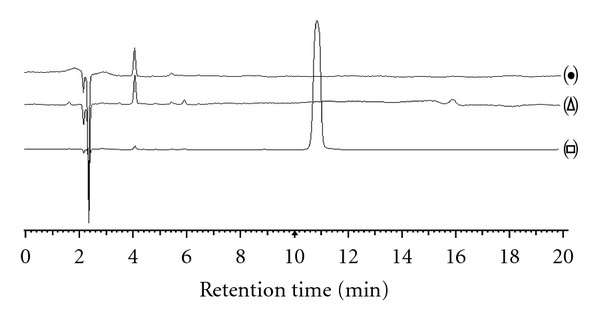
Chromatograms of solvent (•) placebo (∆) and standard solution of CB13 (□).

The specificity of the method was verified by comparing the chromatograms of standard CB13 and those of potential interfering formulation components. The chromatograms obtained in HPLC for the placebo and the solvent do not show any peak with a similar retention time to that of the CB13 (10.88 min ± 10%) (Figure 2). So, it was observed the absence of interferences of the excipients for pharmaceutical preparation, because none of the peaks appears at the same retention time than CB13 peak. Then, it was concluded that the developed method is selective in relation to the excipients of the final preparation. 

Also, tests were performed under three stress conditions (temperature, sun light, and pH) in order to detect the occurrence of possible interfering peaks resulting from degradation of CB13. Furthermore, these tests are regarded as helpful tools in establishing degradation pathways and the inherent stability of the molecule and help invalidating the power of the proposed method for studying the stability of CB13 [26]. According to the areas obtained, the mean degradation value obtained was 7.50%; it can be concluded that CB13 is stable in these conditions. Therefore, the method is selective and suitable for routine work [20].

#### 3.2.2. Precision

Precision expresses the importance that random errors have on the method performance and can be expressed at different levels. In the case of the developed method, precision has been validated for various repeatability studies.

**Instrumental Precision**

The repeatability of the instrumental system was evaluated with replicate injections (*n* = 6) of a single standard preparation (500 *μ*g/mL). In this case an average area of 7895081.83 ± 64499.17 (RSD = 0.82) and an average retention time of 10.86 ± 0.04 min (RSD = 0.34) were obtained. These results indicatedthat the analytical of the instrumental system is in optimum conditions, because the acceptance criterion in analysis of pharmaceutics formulations establishes the limit RSD in 1.5% [23, 24, 33].

**Method Repeatability**

It was determined by using the results obtained in the accuracy tests (in three concentrations levels: low level (50%), middle level (100%), and high level (150%)). The RSD was measured and the values are showed in Table 1. To summarize, RSDs for the six recovery values for levels I, III, and V of the accuracy test are less than 2.0% (acceptance criteria) [27, 29, 33]. For this reason, this study was considered validated*. *

**Intermediate Precision or Reproducibility of Analytical Method**

It was performed on samples containing standard solutions of CB13 by different analysts and different days. The result obtained for the average area was 8097677.88 ± 132650.17 with an RSD value of 1.64. The RSD value of analysis performed was less than 2%, which demonstrates that the method is reproducible, because the introduced variations in the test have no influence on the experimental results [27, 29, 33].

#### 3.2.3. Accuracy

Table 1 shows the results for the six recovery percents obtained for the concentration interval 250–750 *μ*g/mL. The individual values are between 98.04% and 101.91% with an RSD = 1.12%. 

According to the obtained results, it would not be necessary to make any additional statistical test, because ICH for pharmaceutical formulations establishes the recovery percent in accuracy test which must be between 98% and 102%, which is equivalent to ±2.0% of the relative error [27, 33].

#### 3.2.4. Linearity

It was studied in the concentration range of 250–750 *μ*g/mL by calculating the regression equation and the correlation coefficient (*R* ^2^). The equation of the regression lineal obtained corresponds to the following expression:

(2) $$Y=16898*X+317039\;\left(n=6;\;R^{2}=0.9983\right),$$

where *Y* is the  peak area and *X* is the CB13 concentration (*μ*g/mL). 

For the method linearity, the concentration range was the same as that for the system linearity and the equation of the regression line obtained was *Y* = 14303.52∗*X* + 316730.81 with a correlation coefficient *R* ^2^ = 0.9952.

For these studies it was carried out a statistical analysis to ensure a good linearity of the method (ANOVA). The *F* test statistic (*F*) and its corresponding *P* value (significance *F*) certainly indicate an overall goodness of fit for the model (*P* = 2.45 · 10^−11^ for the system linearity and *P* = 1.89 · 10^−14^ for the method linearity). 

#### 3.2.5. Limit of Detection (LOD) and Limit of Quantification (LOQ)

**Figure 3:**
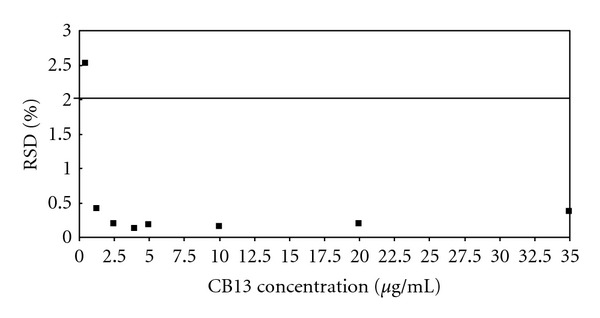
LOQ-LOD obtaining by plotting the area RSD (%) *versus* the CB13 concentrations.

The lowest concentration at which an analyte can be detected (LOD) or quantified with acceptable precision and accuracy (LOQ) was determined by plotting the area RSD (%) *versus* analyte concentration (Figure 3) [34].

The CB13 concentrations used for this study were lower than the end region of the range of the proposed method (0.5, 1.25, 2.5, 4, 5, 10, 20, and 35 *μ*g/mL). The maximum concentration in this study (35 *μ*g/mL) corresponded to a detector signal of around 5% of the minimum area included in the calibration curve.

The first point which does not fulfill the minimum RSD that obeys preset requirements to study reproducibility (2%) corresponds to the LOD. The LOD value was found to be 0.5 *μ*g/mL. The first point which fits into this specified value corresponds to the LOQ, being found at 1.25 *μ*g/mL [35].

The results obtained in this study suggested the employment of a new calibration curve in lower regions of concentrations to quantify minor amounts of CB13; this calibration curve will be useful in some tests such as *in vitro* release studies. For this reason, a new calibration curve was established in the range from 2.5 to 750 *μ*g/mL: *Y* = 17268∗*X* + 190397  (*R* ^2^ = 0,9900)  (*F* = 583.78; *P* = 3.25 · 10^−3^).

#### 3.2.6. Robustness

The evaluation of robustness was based on the percentage recovery and RSD values obtained for different changes in the method analysis and using CB13 solutions at different pH, mobile phase proportion, and temperature and flow rate.  Table 2 shows the recovery percents and the RSD of each changed parameter studied: oven temperature (±2°C); flow rate (±0.100 mL/min); mobile phase proportion (A : B 75 : 25 and A : B 65 : 35) and mobile phase pH (±0.2 units). As is shown in the table, the individual recovery percents obtained in all parameters studied are between 99.33% and 102.87% and the RSD of each one is less than 2%. Thus, the method showed to be robust concerning small but expectable variations of the analysis method [36].

### 3.3. Nanoparticles Characterization and Application of the HPLC Method

The proposed method was applied to study CB13 association with nanoparticulate PLGA carriers produced by three different pathways: SEV-US, SEV-FF, and NPP. These methods are potentially suitable for CB13 encapsulation, a highly lipophilic drug. 

A brief comparative study of nanoparticles production procedures was carried out. The aim of this study was to establish the most appropriate process for CB13 encapsulation into PLGA nanoparticles in terms of particle size, particle size distribution, drug content as well as *in vitro* release profile. For this purpose an RP-HPLC method has been developed and validated. 

Because the preparation and characterization of well-defined sizes of particles remain a challenge, we carried out a brief comparative study gauged by comparing PLGA particles fabricated using two of the main traditional methods for lipophilic drugs (SEV-US and NPP) and by SEV-FF which has been demonstrated be able to produce highly uniform particles [29, 37–39]. Results obtained are summarized in Table 3. As it can be seen, particle size was strongly affected by the synthesis procedure. In SEV-US and SEV-FF it is needed a preemulsion formation. The main difference in these two methods is the energy, applies to form the emulsion. In SEV-US, high energy is applied as ultrasound. This is one of the most employed methods to produce PLGA nanoparticles; the method is economic, simple and provides high performance; nevertheless it presents low control on particle size distribution [38]. For the optimized formulation using this method, particles 420 nm in diameter with VCs higher than 55% were obtained.

In SEV-FF the energy contribution is obtained by liquid pressure (or gas pressure). In this case, a jet is formed; this jet breaks up into drops as a result of a hydrodynamic instability due to tensile strength forces [40]. In general, the main advantages of FF include, among others, (i) particles production occurs under gentle conditions, which makes it suitable for labile compound encapsulation, (ii) it is able to predict the final particle size controlling the particle size distribution, and (iii) it is possible to scale up the rate production when multiple parallel devices are operated [40]. In present work, particles less than one micrometer in diameter were obtained with a very narrow particle size distribution (CV ~ 10%).

In NPP, NPs formation occurs also under mild conditions, which does not require extended shearing/stirring rates, sonication, or very high temperatures. By adding the polymer solution into an aqueous solution a displacement of solvent takes place which is translated into polymer precipitation. NPP appears to be governed by the Marangoni effect, wherein movement in an interface is caused by longitudinal variations of interfacial tension. In such a case, precipitation is driven by (i) solute transfer out of the phase of higher viscosity, which is influenced by high concentration gradients at the interface and (ii) by interfacial tension [41]. Under optimized conditions (polymer concentration, surfactant concentration, addition rate, etc.) it was possible to obtain particles in the nanoscale range with almost narrow particle size distribution (320.73 ± 108.02 nm).

**Figure 4:**
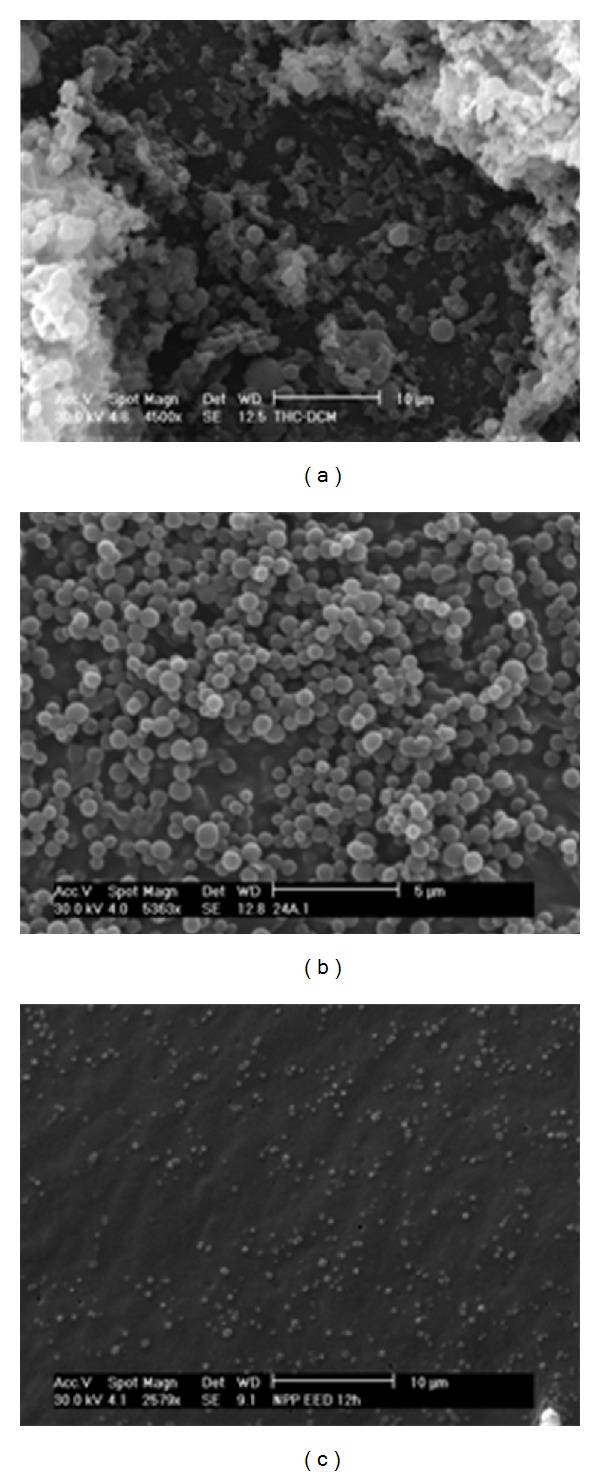
SEM micrographs of CB13-PLGA nanoparticles obtained by three different procedures: (a) SEV-Us, (b) SEV-FF, and (c) NPP. The longitude of the bar indicates a reference value of the dimension of the particle.

In Figure 4 are shown, as examples, SEM micrographs for CB13-PLGA nanoparticles obtained by each method assayed. As it can be seen, spherical and smooth particles were obtained in all cases with narrow-size distribution for SEV-FF and NPP methods. In SEV-FF method especially highly uniform particles were obtained. For instance, in SEV-US method, wide size distribution was obtained and the presence of aggregates was also appreciated. 

Related to nanoparticles surface charge, zeta potential values were measured (Malvern Zetasizer 3000, UK). In all cases, nanoparticles presented zeta potential values slightly negative (around −25 mV) due to chemical structure of PLGA; no influence of nanoparticle synthesis pathway was observed. These results guarantee the stability of nanoparticles in suspension and facilities nanoparticles oral absorption across intestinal membrane [42].

### 3.4. Drug Content in Nanoparticles

The drug loading of the NPs is an important factor in their formulations since high loading implies fewer amounts of the NPs are needed for a given dose of the treatment. The CB13 loading (% w/w) as well as the encapsulation efficiency percentage (EE%) was tested by RP-HPLC. 

In terms of entrapment efficiency (EE%), high values were achieved for all NPs prepared due to the poor solubility of CB13 in the external aqueous phase. In all cases EE was superior to 60%. 

These results indicated a high degree of encapsulation of CB13 into PLGA nanoparticles. Similar results were obtained for lipophilic [13] and hydrophilic [29] molecules in previous works.

### 3.5. *In Vitro* Release Profiles

**Figure 5:**
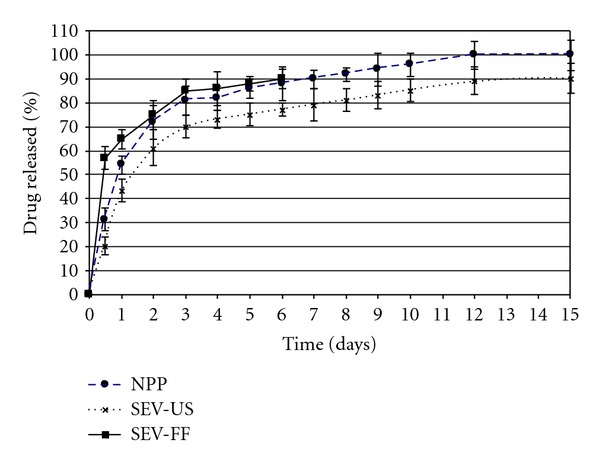
Drug release profile from nanoparticles prepared by three different methods: NPP, SEV-US, and SEV-FF.

The representative CB13* in vitro* release profiles from nanoparticles obtained by three different methods in gastric and intestinal pH-simulated conditions are illustrated in Figure 5. 

This study is used to demonstrate that the developed and validated HPLC method is adequate to characterization of CB13 nanoparticles. In fact, the HPLC method developed is useful to study possible differences in the dissolution behaviour of multiparticulate systems studied

As it can be seen, there is an inverse relationship between release rate and NPs particle size. This has been explained by other authors such as Berkland et al. [43]: they verified that large microspheres degrade more quickly than small microspheres, probably because of an increased accumulation of the acidic products of polymer hydrolysis in large microspheres. Particle size is determined by the synthesis procedure (SEV-FF > SEV-US > NPP).

After 2 h of assay, hydrolysis degradation products of CB13 were not detected and only a well-defined peak appeared on chromatogram, indicating that developed PLGA nanocarrier allows retain CB13 in its structure, so to achieve the stated objectives.

## 4. Conclusions

The proposed HPLC method has been evaluated over the selectivity/specificity, precision, accuracy, linearity, range, limit of detection, limit of quantification, and robustness and proved to be convenient and effective for the quality control of CB13 in PLGA nanoparticles. It has been proved that it was selective, linear between 50% and 150% of the work concentration (500 *μ*g/mL) for CB13, with a correlation coefficient higher than 0.998, exact and precise. Limits of detection and quantification for the drug were 0.5 and 1.25 *μ*g/mL, respectively, and these values are under the lowest expected concentrations in the samples. Moreover, the method has proved that it was robust. 

The HPLC method was applied for CB13 evaluation in PLGA nanoparticles obtained by three different pathways. It was observed that SEV-FF method produced highly uniform particle size although the minimum particle size obtained was in the limit for an efficient oral administration. In NPP, particles obtained presented high drug content with a reasonable EE% and an acceptable particle size and particle size distribution. So, we consider this last synthesis method as the most suitable for CB13-PLGA nanoparticles for oral administration in neuropathic pain treatment.

##  Conflict of Interests

The authors of the paper have no direct financial relationship with any commercial identity mentioned in this paper that could lead to a conflict of interest for any of the authors.

## Figures and Tables

**Table 1:** Results of recovery (%) and RSD (%) for CB13 from standard solutions (*n* = 6) (level I: 250 *μ*g/mL; level II: 375 *μ*g/mL; level III: 500 *μ*g/mL; level IV: 625 *μ*g/mL; level V: 750 *μ*g/mL).

Level	Theoretical mean (*μ*g)	Recovery (%)	R.S.D
I	250.46	101.04	0.54
II	375.48	100.50	1.06
III	500.40	101.47	0.36
IV	625.43	98.86	0.68
V	750.46	101.15	0.46

**Table 2:** Robustness test of proposed method in terms of recovery (%) and RSD (%) for 500 *μ*g/mL CB13 standard (*n* = 3) (MP: mobile phase).

Changes to original method	Theoretical mean (*μ*g)	Peak area	Experimental (*μ*g)	Recovery (%)	RSD
MP proportion (A : B) 65 : 35	500.60	7787498.33	502.83	100.45	0.98
MP proportion (A : B) 75 : 25	500.53	7826118.67	507.95	101.48	1.11
MP pH = 2.70	500.43	7821110.33	507.29	101.37	0.84
MP pH = 3.10	500.50	7811209	505.98	101.09	0.74
Oven temperature −2°C	500.46	7814675	506.44	101.19	1.35
Oven temperature +2°C	500.30	7832734	508.83	101.71	1.04
Flow rate −0.1 mL/min	500.36	7835018,67	509.14	101.75	0.84
Flow rate +0.1 mL/min	500.50	7809528	505.75	101.08	0.87

**Table 3:** Optimized formulations: influence of production method (*n* = 6) (initial amount of CB13 (6% w/w).

Method	D_mean_ ± SD (nm)	ZP (mV)	EE ± SD (%)	Drug loading (%w/w)
SEV-US	420.30 ± 215.43	−29.5 ± 1.9	69.54 ± 0.34	4.172 ± 0.45
SEV-FF	990.61 ± 10.90	−25.6 ± 3.2	92.87 ± 0.96	5.572 ± 0.39
NPP	320.73 ± 108.02	−24.5 ± 2.3	85.69 ± 0.940	5.141 ± 0.67
